# The diagnostic accuracy of ^18^F-FDG PET/CT in diagnosing fracture-related infections

**DOI:** 10.1007/s00259-018-4218-6

**Published:** 2018-12-07

**Authors:** Justin V. C. Lemans, Monique G. G. Hobbelink, Frank F. A. IJpma, Joost D. J. Plate, Janna van den Kieboom, Paul Bosch, Luke P. H. Leenen, Moyo C. Kruyt, Andor W. J. M. Glaudemans, Geertje A. M. Govaert

**Affiliations:** 1 Department of Trauma Surgery, Utrecht University, University Medical Center Utrecht, P.O. Box 85500, 3508 GA Utrecht, The Netherlands; 2 Department of Orthopedics, Utrecht University, University Medical Center Utrecht, Utrecht, The Netherlands; 3 Department of Radiology and Nuclear Medicine, Utrecht University, University Medical Center Utrecht, Utrecht, The Netherlands; 4 Department of Trauma Surgery, University of Groningen, University Medical Center Groningen, Groningen, The Netherlands; 5 Medical Imaging Center, Department of Nuclear Medicine and Molecular Imaging, University of Groningen, University Medical Center Groningen, Groningen, The Netherlands

**Keywords:** Fracture-related infections, ^18^F-FDG PET/CT, Diagnostic performance, Trauma, Infection, Osteomyelitis, Medical imaging, Nuclear imaging, Diagnosis, Diagnostic accuracy

## Abstract

**Purpose:**

^18^F-Fluorodeoxyglucose positron emission tomography (^18^F-FDG PET/CT) is frequently used to diagnose fracture-related infections (FRIs), but its diagnostic performance in this field is still unknown. The aims of this study were: (1) to assess the diagnostic performance of qualitative assessment of ^18^F-FDG PET/CT scans in diagnosing FRI, (2) to establish the diagnostic performance of standardized uptake values (SUVs) extracted from ^18^F-FDG PET/CT scans and to determine their associated optimal cut-off values, and (3) to identify variables that predict a false-positive (FP) or false-negative (FN) ^18^F-FDG PET/CT result.

**Methods:**

This retrospective cohort study included all patients with suspected FRI undergoing ^18^F-FDG PET/CT between 2011 and 2017 in two level-1 trauma centres. Two nuclear medicine physicians independently reassessed all ^18^F-FDG PET/CT scans. The reference standard consisted of the result of at least two deep, representative microbiological cultures or the presence/absence of clinical confirmatory signs of FRI (AO/EBJIS consensus definition) during a follow-up of at least 6 months. Diagnostic performance in terms of sensitivity, specificity, positive predictive value (PPV) and negative predictive value (NPV) was calculated. Additionally, SUVs were measured on ^18^F-FDG PET/CT scans. Volumes of interest were drawn around the suspected and corresponding contralateral areas to obtain absolute values and ratios between suspected and contralateral areas. A multivariable logistic regression analysis was also performed to identify the most important predictor(s) of FP or FN ^18^F-FDG PET/CT results.

**Results:**

The study included 156 ^18^F-FDG PET/CT scans in 135 patients. Qualitative assessment of ^18^F-FDG PET/CT scans showed a sensitivity of 0.89, specificity of 0.80, PPV of 0.74, NPV of 0.91 and diagnostic accuracy of 0.83. SUVs on their own resulted in lower diagnostic performance, but combining them with qualitative assessments yielded an AUC of 0.89 compared to an AUC of 0.84 when considering only the qualitative assessment results (*p* = 0.007). ^18^F-FDG PET/CT performed <1 month after surgery was found to be the independent variable with the highest predictive value for a false test result, with an absolute risk of 46% (95% CI 27–66%), compared with 7% (95% CI 4–12%) in patients with ^18^F-FDG PET/CT performed 1–6 months after surgery.

**Conclusion:**

Qualitative assessment of ^18^F-FDG PET/CT scans had a diagnostic accuracy of 0.83 and an excellent NPV of 0.91 in diagnosing FRI. Adding SUV measurements to qualitative assessment provided additional accuracy in comparison to qualitative assessment alone. An interval between surgery and ^18^F-FDG PET/CT of <1 month was associated with a sharp increase in false test results.

## Introduction

Fracture-related infection (FRI) is a serious complication following trauma surgery and can lead to increased morbidity and high medical costs [1, 2]. Clinical symptoms are not always evident, therefore diagnosing FRI can be challenging. This problem was worsened by the fact that, until recently, there was no uniform definition of FRI [3]. Recently, the AO Foundation (*Arbeitsgemeinschaft für Osteosynthesefragen*) and the European Bone and Joint Infection Society (EBJIS) published a consensus definition comprising confirmatory and suggestive criteria for diagnosing FRI [4]. Medical imaging is considered to be only an adjunct to the diagnosis of FRI (i.e. a suggestive criterion). The reason for this is that the evidence for its accuracy in diagnosing FRI is limited. Moreover, such evidence as is available was obtained mainly from studies investigating other causes of bone infection such as diabetic foot infection, periprosthetic joint infection (PJI) and haematogenous osteomyelitis [5]. Most previous studies on diagnostic imaging of FRI have been hampered by small patient cohorts, unclear reference standards and heterogeneous patient populations [5, 6]. Recently, our group found that white blood cell (WBC) scintigraphy has a high accuracy (0.92) when diagnosing FRI [7]. To compare imaging modalities, we used the same study design to evaluate the diagnostic performance of ^18^F-fluorodeoxyglucose positron emission tomography/computed tomography (^18^F-FDG PET/CT).

The aims of the current study were:

1. To establish the performance of qualitative assessment of ^18^F-FDG PET/CT scans in diagnosing FRI
2. To establish the performance of standardized uptake values (SUVs) from ^18^F-FDG PET/CT in diagnosing FRI and to determine their optimal associated cut-off values
3. To determine which variables are independent predictors of a false positive (FP) or false negative (FN) ^18^F-FDG PET/CT test result in patients with suspected FRI

## Materials and methods

### Ethical approval

Due to the observational nature of this study the need for informed consent was waived by the Medical Ethics Review Committee (METC) of the University Medical Center Utrecht (METC 17-475).

### Study design and eligibility criteria

This was a two-centre, retrospective cohort study that included patients from two large level-1 trauma centres in The Netherlands: the University Medical Center Utrecht and the University Medical Center Groningen. All consecutive patients undergoing ^18^F-FDG PET/CT for diagnosing (or excluding) FRI between January 2011 and November 2017 were eligible for inclusion. FRI was considered as either an infection following an open fracture (irrespective of type of treatment), an infection following fracture surgery, or an infection following instrumented fusion for spinal fractures. Skeletally immature patients (<16 years old) and patients undergoing ^18^F-FDG PET/CT for reasons other than diagnosing FRI (such as PJI, nontraumatic osteosyntheses or haematogenous osteomyelitis) were excluded. Patients in whom the reference test did not meet the criteria for validity, as described in the section Reference test, were also excluded.

### Index test

The index test was the ^18^F-FDG PET/CT scan. Scanning protocols were similar in both centres. Scans were acquired approximately 60 min after intravenous administration of 2–3 MBq/kg ^18^F-FDG according to existing European Association of Nuclear Medicine (EANM) guidelines for ^18^F imaging [8]. Scans were acquired on either a Biograph mCT 64-slice or a Biograph mCT 40-slice PET/CT system (Siemens, Knoxville, TN, USA). No metal artefact reduction algorithm was used in either centre.

**Fig. 1:**
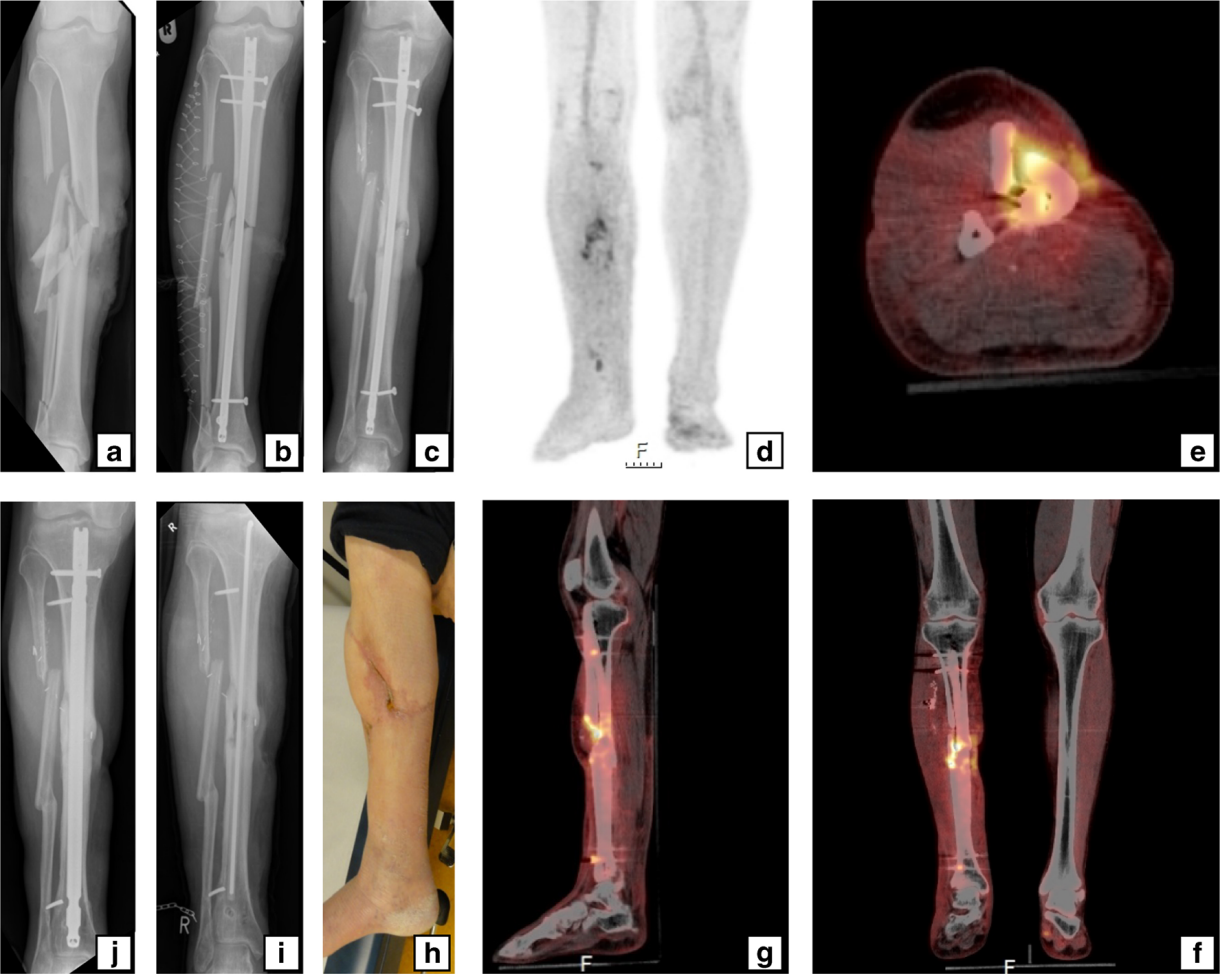
A 59-year-old man sustained a right-sided Gustilo grade IIIB open crural fracture (**a**) which was treated with intramedullary nailing and a fasciotomy (**b**). After several soft-tissue debridement procedures, the remaining soft tissue defect was eventually closed with a free musculocutaneous flap. After 20 months, there was a non-union with “autodynamization” of the intramedullary nail, demonstrated by broken interlocking screws (**c**). The ^18^F-FDG PET image (**d**) shows increased uptake around the fracture site in the tibial shaft and around the proximal and distal screws. The hybrid ^18^F-FDG PET/CT images (**e** axial, **f** coronal, **g** sagittal) localize the suspected fracture-related infection (FRI) not only to the fracture site but also to the surrounding bone of the tibia around the fracture site which corresponds to the unstable scar overlapping the area of the non-union (**h**). The intramedullary nail was removed, the tibia was reamed, the fracture site was debrided and an in-house, custom-made antibiotic nail was inserted (**I**). FRI was confirmed by microbiological cultures and the patient was subsequently treated with antibiotics. One year after exchange nailing, fracture healing was successful (**j**)

After anonymization, the scans were independently reassessed by two experienced nuclear medicine physicians (M.G.G.H. and A.W.J.M.G.). Both the attenuation-corrected images and the images without attenuation correction were reviewed. Both nuclear medicine physicians were blinded to the reference test result. Nuclear imaging signs were documented for each of the scans on a case report form (CRF). These signs included uptake location, uptake pattern (multifocal, heterogeneous, diffuse homogeneous), uptake grade (*0:* no uptake, *1:* higher uptake in the side with suspected infection than in the contralateral side, *2:* much higher uptake in the side with suspected infection than in the contralateral side), involvement of osteosynthesis material, and soft-tissue and bone involvement. Disagreements were resolved through discussion until consensus was reached. A clinical case example of the use of ^18^F-FDG PET/CT for diagnosing FRI is provided in Fig. 1.

For semiquantitative analysis, SUVs were also measured on ^18^F-FDG-PET/CT scans reconstructed according to EANM EARL protocols. SUVs correspond to the extent of ^18^F-FDG uptake and consequently reflect cellular glucose metabolism. Because glucose metabolism is increased in infected tissues, higher SUVs correspond to a greater risk of FRI than lower SUVs [9]. SUVs were determined by drawing a spherical volume of interest (VOI) on both the target area with suspected infection and a corresponding anatomical reference area on the contralateral side. Additionally, a VOI was drawn on nearby muscle for background comparison. For all VOIs, both SUV_max_ (single-pixel value) and SUV_peak_ (average value in a high-uptake part of the VOI) were calculated. For both SUV_max_ and SUV_peak_, the ratios between the suspected infected side and the contralateral side were also calculated (SUV_maxratio_ and SUV_peakratio_). To correct for background ^18^F-FDG uptake, ratios between the SUVs of the suspected infected site and the SUVs of nearby muscles (SUV_maxmuscleratio_ and SUV_peakmuscleratio_) were calculated. These data were reported in a separate CRF as continuous measurements. All SUV measurements were corrected for body weight and blood glucose level and were performed with *syngo*.via software (Siemens Healthineers, Forchheim, Germany).

### Reference test

The final diagnosis of FRI (reference test) was based on the outcome of medical microbiological (MMB) culture results in patients with surgical intervention, or – if unavailable – on clinical follow-up of at least 6 months. Because this study involved the retrospective analysis of culture results obtained in an era when no uniform culturing protocol existed, strict criteria for judging the validity of the reference test were applied. All MMB results were judged by an experienced trauma surgeon on their ability to correctly detect FRI. The microbiological results from swabs and cultures of fistulas were disregarded due to relatively low accuracy [10–12]. The MMB results were only considered representative if cultures of at least two surgically obtained deep-tissue samples from the site of suspected infection were available. A positive FRI result was defined as at least two positive representative MMB cultures with the same microorganism according to the microbiological criteria outlined in the AO/EBJIS consensus definition [4]. FRI during clinical follow-up was defined according to the clinical confirmatory criteria of the AO/EBJIS consensus definition as any wound breakdown, purulent drainage or the presence or development of a sinus tract (communicating with the implant material) [4]. If culture results were negative but confirmatory criteria for FRI were met (e.g. pus, fistula) peroperatively when cultures were taken, FRI was deemed to be present (and the culture result was considered to be erroneous). Culture-negative FRIs are known to be caused by bacteria with low virulence such as coagulase-negative *Staphylococcus* species [13].

### Statistical analyses

To assess the diagnostic performance of the ^18^F-FDG PET/CT scan, the number of true-positive (TP), FP, true-negative (TN) and FN test results were obtained. From this, sensitivity, specificity, positive predictive values (PPV), negative predictive values (NPV), positive and negative likelihood ratios and diagnostic odds ratios with 95% confidence intervals (CI) were calculated. A sensitivity analysis was performed including only the first scan in each patient to determine whether selection bias of patients undergoing multiple scans may have contributed to differences in diagnostic parameters.

All SUVs were compared between groups using Student’s *t* test (if normally distributed) or the Mann-Whitney *U* test (if not normally distributed). Normality of the data was determined by visual inspection of normality plots. The sensitivity and specificity of the separate SUV measurements were plotted as receiver operating characteristic (ROC) curves and for each curve, the area under the curve (AUC) was calculated. The Q-point on each curve (i.e. the point at which sensitivity and specificity were maximized) was determined and the associated cut-off value was extracted. In addition, an ROC curve was plotted combining the diagnostic performance of SUV measurements with the performance of qualitative assessment. The difference between the ROC curve from the combined analysis and the ROC curve with only the qualitative assessment was analysed using the test described by DeLong et al. [14]. To ensure that this test was appropriately applied in this situation of nested models, we investigated whether the added variable “combined SUV measurements” in the combined model was independently associated with the outcome [15].

Consequently, a backward stepwise multivariable logistic regression analysis was performed to determine which variables were independent predictors of a false (i.e. FP or FN) test result. Removal testing was performed with the probabilities of the likelihood ratio statistic based on the maximum partial likelihood estimates. Multiple variables suggested in the literature to influence ^18^F-FDG PET/CT accuracy were included in the model [16]. The variables entered were: interval between the last operative procedure (or date of trauma if no operation was performed) and the ^18^F-FDG PET/CT scan (ordinal; <1 month, between 1 and 6 months and >6 months), body mass index (continuous), presence of diabetes mellitus (dichotomous), smoking history (dichotomous), nonsteroidal antiinflammatory drug (NSAID) use at the time of ^18^F-FDG PET/CT (dichotomous) and antibiotic use at the time of ^18^F-FDG PET/CT (dichotomous). Using the final model, the probabilities of false test results were obtained (with 95% CIs) for the different variables. Additionally, the diagnostic performance of qualitative assessment was calculated excluding scans with a high risk of a false test result. All statistical analyses were performed with SPSS Statistics version 25.0 (IBM Corp., Armonk, NY).

## Results

In the study period, 154 patients underwent 176 ^18^F-FDG PET/CT scans for suspected FRI. The reference test was not performed in 18 patients and these patients were excluded. Two ^18^F-FDG PET/CT scans in skeletally immature patients were also excluded. A total of 135 patients who underwent 156 ^18^F-FDG PET/CT scans were ultimately included. The patient characteristics are summarized in Table 1. The fracture specifics are presented in Table 2, and the types of index operation in Table 3.

**Table 1:** Baseline characteristics

Characteristic	Value
Age (years), mean (range)	46.7 (16–76)
Sex (male), *n* (%)	112 (71.8)
Body mass index (kg/m^2^), mean (range)	27.1 (15.3–48.1)
ASA score, *n* (%)	
1	58 (37.2)
2	73 (46.8)
3	10 (6.4)
4	1 (0.6)
Unknown	14 (9.0)
Injury severity score, *n* (%)	
<16	91 (58.3)
≥16	58 (37.2)
Unknown	7 (4.5)
Comorbidities/risk factors at time of ^18^F-FDG PET/CT, *n* (%)	
Diabetes mellitus	16 (10.3)
Psychiatric disease	15 (9.6)
Obesity	31 (19.9)
Hypothyroidism	4 (2.6)
Hypertension	19 (12.2)
Tobacco use	63 (40.4)
Alcohol abuse	11 (7.1)
Drug abuse	9 (5.8)
NSAID use	34 (21.8)
Corticosteroid use	3 (1.9)
Antibiotic use	35 (22.4)

*ASA* American Society of Anesthesiologists, *NSAID* nonsteroidal antiinflammatory drug

**Table 2:** Fracture characteristics

Classification	Number (%) of scans
AO classification	
1: Humerus fractures	5 (3.2)
13: Distal	1 (0.6)
15: Clavicle	4 (2.6)
2: Radius/ulna fractures	8 (5.1)
21: Proximal	3 (1.9)
22: Diaphyseal	3 (1.9)
23: Distal	2 (1.3)
3: Femur fractures	25 (16.0)
31: Proximal	1 (0.6)
32: Diaphyseal	18 (11.5)
33: Distal	6 (3.8)
4: Tibia/fibula fractures	88 (56.4)
41: Proximal	12 (7.7)
42: Diaphyseal	48 (30.8)
43: Distal	16 (10.3)
44: Malleolar	12 (7.7)
5: Spine fractures	14 (9.0)
A: Compression injury	9 (5.8)
B: Distraction injury	1 (0.6)
C: Dislocation injury	3 (1.9)
Unknown	1 (0.6)
6: Pelvis/sacrum fractures	5 (3.2)
8: Foot fractures	11 (7.1)
81: Talus	3 (1.9)
82: Calcaneus	6 (3.8)
83: Navicular	1 (0.6)
Unknown	1 (0.6)
Gustilo-Anderson classification	
Closed fractures	68 (43.6)
Open fractures	76 (48.7)
Type I	13 (8.3)
Type II	11 (7.1)
Type IIIA	20 (12.8)
Type IIIB	6 (3.8)
Type IIIC	3 (1.9)
Unknown	23 (14.7)
Unknown	12 (7.7)

*AO* Arbeitsgemeinschaft für Osteosynthesefragen

**Table 3:** Index procedures

Procedure	Number (%) of scans
Operative	150 (96.2)
Plate	53 (34.0)
Screw(s)	16 (10.3)
Intramedullary nail	35 (22.4)
Arthrodesis (including spinal fusion)	14 (9.0)
Amputation	1 (0.6)
External fixator	31 (19.9)
followed by:	
Plate	17 (10.9)
Screw	1 (0.6)
Intramedullary nail	5 (3.2)
Conservative	2 (1.3)
Unknown	6 (3.8)
Closed reduction/conservative	5 (3.2)
Unknown	1 (0.6)

For 67 ^18^F-FDG PET/CT scans (43%), a representative MMB culture result was available. These scans were obtained from patients with a median clinical follow-up of 13 months (IQR 20 months), 33 of these scans (49%) were obtained from patients that had a MMB culture-confirmed FRI. *Staphylococcus* species were most commonly cultured (Table 4). In 11 patients, culture results were negative but there were peroperative confirmatory signs of FRI, including purulent drainage, wound breakdown or a fistula communicating with implant material. These patients were scored as positive for FRI.

**Table 4:** Microbiological findings in 33 patients with MMB culture-confirmed FRI in relation to the ^18^F-FDG PET/CT result

Species cultured	^18^F-FDG PET/CT result	
	True-positive (*N* = 31)	False-negative (*N* = 2)
*Staphylococcus aureus*	12	1
Coagulase-negative *Staphylococcus* spp.	10	
*Streptococcus* spp.	4	
*Corynebacterium* spp.	2	
*Enterococcus* spp.	4	
*Finegoldia magna*		1
*Actinomyces neuii*	1	
*Propionibacterium acnes*	1	
*Pseudomonas aeruginosa*	4	
*Escherichia coli*	2	1
*Enterobacter cloacae*	2	
*Serratia marcescens*	1	
*Fusobacterium gonidiaformans*	1	
*Bacteroides thetaiotaomicron*	1	
*Proteus vulgaris*	1	
*Klebsiella oxytoca*	1	
*Morganella morganii*	1	
*Bacteroides fragilis*	1	
Polymicrobial	11	1

For 89 ^18^F-FDG PET/CT scans (57%), representative MMB culture results were not available. These scans were obtained from patients with a median clinical follow-up of 16 months (IQR 23 months), 18 of these scans were obtained from patients that showed clinical confirmatory signs of FRI, the remainder of these patients had an uneventful clinical follow-up. The 71 remaining patients had an uneventful clinical follow-up. In total, 62 patients were diagnosed with FRI. In 55 of these 62 patients, ^18^F-FDG PET/CT was positive for FRI (TP). In 75 of 94 patients negative for FRI, ^18^F-FDG PET/CT correctly ruled out an FRI (TN). The ^18^F-FDG PET/CT result was FP in 19 patients and FN in 7 patients. Thus, ^18^F-FDG PET/CT showed a diagnostic sensitivity of 0.89 (95% CI 0.78–0.95), specificity of 0.80 (95% CI 0.70–0.87), PPV of 0.74 (95% CI 0.66–0.81), NPV of 0.91 (95% CI 0.84–0.96), positive likelihood ratio of 4.39 (95% CI 2.91–6.62), negative likelihood ratio of 0.14 (95% CI 0.07–0.29), and diagnostic odds ratio of 31.0 (95% CI 12.2–78.9). The accuracy of ^18^F-FDG PET/CT for diagnosing FRI was 0.83 (95% CI 0.77–0.89). The sensitivity analysis including only the first ^18^F-FDG PET/CT scan in each patient (*N* = 135) resulted in similar diagnostic parameters: sensitivity 0.91 (95% CI 0.80–0.97), specificity 0.81 (95% CI 0.70–0.89), PPV 0.77 (95% CI 0.68–0.84), NPV 0.93 (95% CI 0.84–0.97) and diagnostic accuracy 0.85 (95% CI 0.78–0.91).

### Semiquantitative measurements

Semiquantitative SUV measurements are presented in Table 5. Patients with FRI had a median SUV_max_ of 5.9 (IQR 3.5) and median SUV_peak_ of 4.7 (IQR 2.4) in the area with suspected infection. Patients without FRI had a median SUV_max_ of 3.2 (IQR 2.5) and a median SUV_peak_ of 2.6 (IQR 1.9) in the area initially suspected of infection. The differences in both SUV_max_ and SUV_peak_ between the groups were significant (both *p* < 0.001). In patients with FRI, the SUV ratios for the area with suspected infection in relation to the contralateral area were 3.0 (IQR 2.1) for SUV_max_ and 2.9 (IQR 2.0) for SUV_peak_. In patients without FRI, the ratios were 1.9 (IQR 1.4) and 1.8 (IQR 1.4), respectively. Both ratios were significantly different between patients with and without FRI (*p* < 0.001). In patients with FRI, the SUV ratios for the area with suspected infection in relation to nearby muscle were 6.4 (IQR 4.9) for SUV_max_ and 5.5 (IQR 3.6) for SUV_peak_. In patients without FRI, the ratios were 3.5 (IQR 3.0) and 3.3 (IQR 2.9), respectively. These ratios were also significantly different between patients with and without FRI (*p* < 0.001).

**Table 5:** Semiquantitative SUV measurements in relation to the presence of FRI

	All 18F-FDG PET/CT scans (*N* = 155)^a^	18F-FDG PET/CT scans positive for FRI (*N* = 61)^a^	18F-FDG PET/CT scans negative for FRI (*N* = 94)	*p* value
^18^F-FDG dose (MBq)	193.0 (77.0)	199.0 (132.0)	192.0 (70.0)	0.287
Blood glucose (mmol/l)	5.6 (1.0)	5.7 (0.9)	5.5 (1.1)	0.241
SUV_max_				
Infection location	4.2 (3.4)	5.9 (3.5)	3.2 (2.5)	<0.001
Contralateral location	1.7 (0.7)	1.8 (0.9)	1.7 (0.7)	0.039
Ratios^b^				
Infection/Contralateral	2.1 (1.8)	3.0 (2.1)	1.9 (1.4)	<0.001
Infection/Muscle	4.6 (3.9)	6.4 (4.9)	3.5 (3.0)	<0.001
SUV_peak_				
Infection location	3.5 (2.7)	4.7 (2.4)	2.6 (1.9)	<0.001
Contralateral location	1.4 (0.7)	1.5 (0.7)	1.4 (0.7)	0.070
Ratios^b^				
Infection/Contralateral	2.1 (1.8)	2.9 (2.0)	1.8 (1.4)	<0.001
Infection/Muscle	4.1 (3.4)	5.5 (3.6)	3.3 (2.9)	<0.001

Data are presented as medians (IQR)

*FRI* fracture-related infection

^a^SUV measurements could not be retrieved in one patient for technical reasons.

^b^Ratios were calculated by dividing the SUV of the suspected infected area by the SUV of the contralateral area/nearby muscle; a value of >1 signifies higher uptake in the suspected infected area.

**Fig. 2:**
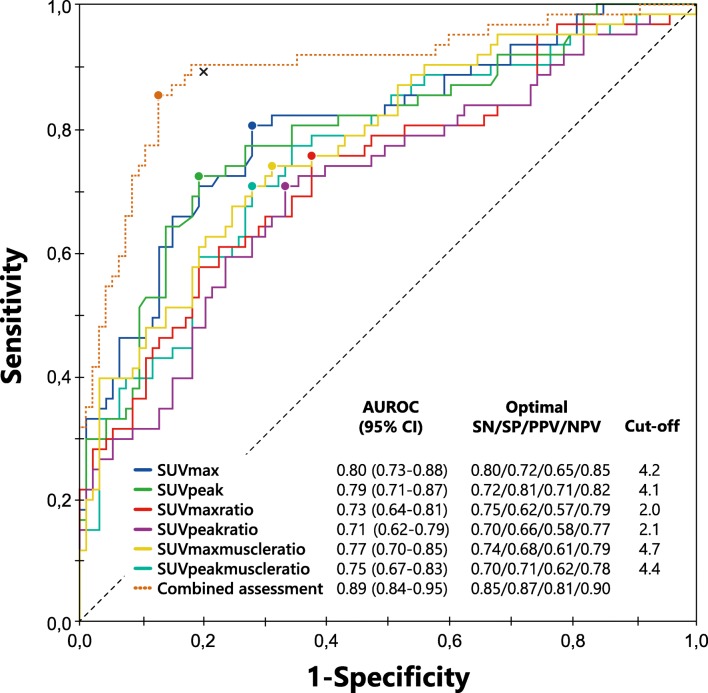
Receiver operating characteristics (ROC) curves for the semiquantitative SUV measurements analysed separately and in combination with the qualitative ^18^F-FDG PET/CT assessment data. The *circles* on the curves represent the Q-points (i.e. the optimum between sensitivity and specificity at a specific cut-off value). The *cross* represents the sensitivity and specificity of the qualitative ^18^F-FDG PET/CT assessment. This point is higher than any of the Q-points for the semiquantitative measurements alone. The area under the curve for the combined qualitative and semiquantitative assessment (dotted line) is 0.89, higher than the areas under the curve for the semiquantitative measurements analysed separately and also higher than the AUC of the qualitative assessment alone. *AUROC* area under the receiver operator characteristics curve, *SN* sensitivity, *SP* specificity, *PPV* positive predictive value, *NPV* negative predictive value

ROC curves for the semiquantitative SUV data are shown in Fig. 2. The areas under the curve were 0.80 (95% CI 0.73–0.88) for SUV_max_, 0.73 (95% CI 0.64–0.81) for SUV_maxratio_ and 0.77 (95% CI 0.70–0.85) for SUV_maxmuscleratio_. Optimal sensitivity and specificity for SUV_max_ were 0.80 and 0.72 at a cut-off value of 4.2. The PPV and NPV for SUV_max_ at this cut-off value were 0.65 and 0.85, respectively. For SUV_maxratio_, sensitivity was 0.75 and specificity was 0.62 at a cut-off value of 2.0, and for SUV_maxmuscleratio_, sensitivity was 0.74 and specificity was 0.68 at a cut-off value of 4.7. The diagnostic parameters and associated cut-off values for SUV_peak_ were similar to those for SUV_max_ and are also shown in Fig. 2.

Combining the SUV measurement data with the qualitative assessment of ^18^F-FDG PET/CT scans in a separate ROC curve yielded an AUC of 0.89 (95% CI 0.84–0.95) and a diagnostic accuracy of 0.86 (sensitivity 0.85, specificity 0.87, PPV 0.81, NPV 0.90), in contrast to an AUC of 0.84 (95% CI 0.78–0.91) and a diagnostic accuracy of 0.83 for the qualitative assessment on its own. The added explanatory variable “combined SUV measurements” was independently associated with the presence/absence of FRI and comparison of the ROC curves was deemed appropriate. The AUC of the combined assessment was 0.05 (95% CI 0.01–0.09) greater than the AUC of the qualitative assessment alone (*p* = 0.007).

### Characteristics of patients with false-negative/false-positive results

Seven patients were included with a FN test result. Two patients had positive intraoperative cultures, while five patients showed confirmatory signs peroperatively or during the 6-month follow-up. Two patients had (low-grade) infection of a non-union (both ankle fractures). Another patient (with two scans) showed peroperative signs of FRI in the tibia (infected tissue and pus) despite microbiological cultures remaining negative. There were 19 patients with a FP test result. These included two patients with a lower arm fracture, two with a femoral fracture, two with a tibial plateau fracture, seven with a lower leg fracture, two with an ankle fracture, two with a talar fracture and two with a spinal fracture. Eight patients had a negative intraoperative culture, 11 had no cultures taken but showed no signs of FRI during the 6-month follow-up. Five patients (26%) with a FP result underwent surgery during the week before the ^18^F-FDG PET/CT scan (one with a tibial fracture, one with a talar fracture, one with an ankle fracture, and two with a tibial plateau fracture). These scans were performed to determine if the FRI had receded or was still advancing in patients who underwent surgery for suspected FRI shortly before the scan.

### Predictors of a false test result

The most important predictor of a false test result was an interval of <1 month between the last operative procedure and the ^18^F-FDG PET/CT scan (*B* = 2.461, intercept −2.615). The associated absolute predicted risk of a false result with this variable was 46% (95% CI 27–66%) compared with an absolute predicted risk of the reference group (with an interval of 1–6 months) of 7% (95% CI 4–12%). In patients with an interval of >6 months, the absolute risk was 17% (95% CI 10–29%). The test result was erroneous in 6 of 14 patients (42.9%) undergoing ^18^F-FDG PET/CT within 1 month (FP in all six patients). The rate of erroneous test results reduced to 8.9% (4 of 45 patients) in those with an interval between 1 and 6 months, and showed a slight increase to 16.8% (16 out of 95 patients) in those with an interval of more than 6 months. Omitting the results from the early ^18^F-FDG PET/CT scans (performed within 1 month of surgery) led to an increase in diagnostic accuracy of the qualitative assessment to 0.86 (95% CI 0.79–0.91) with a sensitivity and specificity of 0.88 (95% CI 0.76–0.95) and 0.85 (95% CI 0.76–0.92), respectively.

## Discussion

The current study showed that qualitative assessment of ^18^FDG PET/CT scans has good performance in diagnosing FRI with a diagnostic accuracy of 0.83 (95% CI 0.77–0.89) and an AUC of 0.84 (95% CI 0.78–0.91). The NPV (0.91) was notably higher than that of most other imaging modalities, and makes ^18^FDG PET/CT an excellent tool for use in patients with chronic or low-grade infections [5]. Combining the results of qualitative assessment and SUV measurements resulted in an even higher diagnostic accuracy (0.86) and an AUC of 0.89 (95% CI 0.84–0.95), which shows that including SUV measurements increased diagnostic accuracy, although the increase was relatively small.

The sensitivity and specificity rates found in this study are in line with those found in other studies on the accuracy of ^18^F-FDG PET/CT in diagnosing FRI [5, 9]. However, this study also included semiquantitative measurements and used strict ^18^F-FDG PET/CT assessment and reference test criteria (based on the recently released AO/EBJIS consensus definition of FRI) [4]. It also included the largest series to date of patients with suspected FRI undergoing hybrid ^18^F-FDG PET/CT imaging. One systematic review and meta-analysis investigating the accuracy of different imaging modalities for diagnosing chronic osteomyelitis showed higher diagnostic accuracy of ^18^F-FDG PET with a pooled sensitivity of 0.96 and a specificity of 0.91 [6]. That study, however, included only studies published before 2003 and investigated only ^18^F-FDG PET without fusion CT images, which is now rarely used following the advent of ^18^F-FDG PET/CT scanners. In addition, reference test criteria were unclear in some of the studies reviewed and the studies included few patients and a relatively large number of spinal ^18^F-FDG PET/CT scans. A more recent systematic review found that the sensitivities and specificities of ^18^F-FDG-PET/CT in diagnosing FRI ranges between 0.86–0.94 and 0.76–1.00, respectively [5]. These results, as well as the methodology used (patient population and reference standard) are comparable to those used in our study.

There is only limited research on the accuracy of quantification in diagnosing FRI. A recent study on the accuracy of SUV measurements from ^18^F-FDG PET/CT for diagnosing FRI found a sensitivity of 0.65 and specificity of 0.77 at a SUV_max_ cut-off value of 4.0 [17]. These values are lower than those published previously for qualitative assessment of ^18^F-FDG PET/CT scans [5]. The reason for this could be that the previous SUV measurement study used only ^18^F-FDG PET/CT to differentiate between infected non-unions and aseptic non-unions. In both circumstances, increased bone metabolism will often be found, and thus differences between ^18^F-FDG uptake will be limited. The cut-off value of 4.0 used in the previous study is similar to the SUV_max_ cut-off value found in the current study (4.2). Unfortunately, the validity of the results is difficult to compare between our study and the previous study, because it is unclear whether the standardized EARL scanning protocols were used in the latter [18]. Additionally, only semiquantitative measurements, and no qualitative criteria (such as uptake pattern and grade) for diagnosing FRI were used. SUV measurements do not take into account the activity pattern and uptake location, and can be positive as a consequence of both bone healing and/or non-union. Therefore, using only semiquantitative data might lead to misclassification of some patients. This is supported by the results of our study, in which the diagnostic accuracy of the qualitative assessment by the nuclear medicine physicians was higher than the accuracy when using SUVs alone. This phenomenon was also seen in a large study of patients with FRI which demonstrated a diagnostic accuracy of 0.82 with qualitative assessment of ^18^F-FDG PET(/CT) scans and a lower accuracy with only semiquantitative measurements (SUV_max_ sensitivity 0.69, specificity 0.66 using a cut-off value of 3.9) [9]. Another study investigating SUVs in histologically proven culture-positive and culture-negative patients with FRI showed that SUVs in both groups of patients were similar (SUV_max_ 3.73 in culture-positive patients, 2.81 in culture-negative patients) [19]. The findings of these studies, as well as those of the current study, add to the mounting evidence that semiquantitative measurements can be used as additional diagnostic tools for diagnosing FRI.

WBC scintigraphy has been more thoroughly investigated as an imaging modality for diagnosing FRI. Our previous study of WBC scintigraphy found a diagnostic accuracy of 0.92, which is higher than the diagnostic accuracy found in the current study for ^18^F-FDG PET/CT [7]. However, ^18^F-FDG PET/CT does have several advantages over WBC scintigraphy. First, there is no need for manipulation of leukocytes, which is a labourious and expensive part of WBC scintigraphy [20]. Second, ^18^F-FDG PET/CT can be performed much more quickly (1 h following radionuclide injection) and takes only one scanning session, as opposed to WBC scintigraphy, which takes at least two scans (4 h and 20–24 h after radionuclide injection) on two consecutive days [20]. Third, WBC scintigraphy has lower accuracy when used for diagnosing infections in the axial skeleton due to physiological uptake in the bone marrow, while ^18^F-FDG PET/CT does not have this limitation [16]. ^18^F-FDG PET/CT has the disadvantage that implants negatively affect diagnostic accuracy, although in some studies, this effect has not been shown [5, 9]. With the recent onset of several techniques for metal artefact reduction in the newest generation PET/CT camera systems, the diagnostic performance of both qualitative assessment and quantification in patients with an implant and suspected FRI can probably be improved further. Ultimately, both imaging modalities have their specific advantages and limitations and although ^18^F-FDG PET/CT has lower accuracy than WBC scintigraphy, its advantages in terms of logistics and patient comfort make it a good alternative to WBC scintigraphy as the first nuclear imaging modality to perform when diagnosing FRI. Thus, both modalities can be used to diagnose FRI depending on physician/hospital preference, financial considerations, and/or experience with either technique.

We found that performing the ^18^F-FDG PET/CT scan <1 month following surgery was correlated with a FP ^18^F-FDG PET/CT result. It is known that operative procedures cause tissue damage and inflammation/regeneration, and affected tissue shows increased uptake of ^18^F-FDG, especially when the interval between the ^18^F-FDG PET/CT and surgery is short [16]. Five of the FP ^18^F-FDG PET/CT scans were performed within a week of an operative procedure. Both nuclear medicine physicians reassessing these scans for this study agreed that in some of these scans, inflammation due to surgery was indistinguishable from FRI. We conclude that ^18^F-FDG PET/CT should therefore not be performed as a diagnostic tool within a month of surgery. If (per protocol) early (<1 month after surgery) ^18^F-FDG PET/CT scans for suspected FRI are no longer performed, diagnostic accuracy can be expected to improve, in this study exclusion of such early scans led to an increase in accuracy from 0.83 to 0.86.

The strengths of the current study are the large cohort size, and the fact that a robust, standardized and repeatable scan assessment was performed by two independent nuclear medicine physicians (one from each hospital) who were blinded to the reference standard. We also used strict reference standard criteria to determine whether FRI was present or not, based on the recently published FRI consensus definition [4]. Finally, the addition of SUV measurements and SUV analysis provided additional insight into its merits and its performance compared to standard qualitative assessments.

The limitations of the current study include its retrospective design, with the associated risks of selection- and differential misclassification bias. Patients were recruited in two different teaching hospitals, thus there may have been differences in the diagnostic work-up and treatment of FRI, as each hospital has its own standard of care. Also, in some patients, FRI had already been diagnosed and the ^18^F-FDG PET/CT scans were used for treatment follow-up. This mainly occurred at the beginning of the study period; since then, stricter protocols have been adopted, which aim to standardize both ^18^F-FDG PET/CT indications and microbiological culture acquisition and treatment regimens. Finally, it is important to remember that the combined assessment by two nuclear medicine specialists might have led to a higher diagnostic accuracy than can be obtained in the normal clinical situation, in which only one nuclear medicine physician reviews a scan. Further prospective studies to compare different imaging modalities for diagnosing FRI are warranted.

### Conclusion

The results of the study can be summarized as follows:

1. Qualitative assessment of ^18^F-FDG PET/CT scans has good accuracy (0.83) for diagnosing FRI, with an excellent NPV of 0.91.
2. SUV measurements provide additional diagnostic accuracy when added to qualitative assessment of ^18^F-FDG PET/CT scans.
3. ^18^F-FDG PET/CT should not be performed for diagnosis within a month of surgery.
